# Treatment of Wounds

**Published:** 1900-03

**Authors:** 


Treatment of Wounds.—Its principles and practice, general and special. By L. S. Pilcher, A. M., M. D., Surgeon to the Methodist-Episcopal Hospital in New York, etc., etc. One volume, 8 vo., 466 pages. Profusely illustrated. Muslin, $3.00, net. William Wood & Company.

The author, in 1883, wrote a book on "The Treatment of Wounds," which appeared in "Wood's Library of Standard Authors." It became a popular work, being an advocate of antiseptics for everything. The general plan of the book has been followed in the new volume, but that is all. The advancement in the treatment of wounds has been so great that the author was practically compelled to write a new book in order to present the present status of surgery.

The book starts off, in Part I, with a description of the principles of wound treatment. Then follows a general discussion of wounds, their immediate effects, their repair, inflammation, the relation of micro-organisms to wound disturbances, asepsis and antisepsis, wound cleanliness, wound disinfection and antiseptics. Section second is devoted to wound treatment, the arrest of hemorrhage, wound cleanliness, antiseptic dressings, gangrene, erysipelas and septicemia. Part II is devoted to a consideration of the various kind of wounds—subcutaneous, incised, contused, lacerated and gunshot wounds; wounds of muscles, tendons, nerves, blood vessels and bones; then wounds of location—the head, neck, throat, abdomen and the extremities.

Each subject is discussed thoroughly and in a manner to be of the greatest benefit to the general practitioner. T. J. B.

